# Strength Training for Arthritis Trial (START): design and rationale

**DOI:** 10.1186/1471-2474-14-208

**Published:** 2013-07-15

**Authors:** Stephen P Messier, Shannon L Mihalko, Daniel P Beavers, Barbara J Nicklas, Paul DeVita, J Jeffery Carr, David J Hunter, Jeff D Williamson, Kim L Bennell, Ali Guermazi, Mary Lyles, Richard F Loeser

**Affiliations:** 1 Department of Health and Exercise Science, Wake Forest University, Winston-Salem, NC 27109, USA; 2 Department of Biostatistical Sciences, Wake Forest School of Medicine, Winston-Salem, NC, USA; 3 Section on Gerontology and Geriatric Medicine, Wake Forest School of Medicine, Winston-Salem, NC, USA; 4 Department of Exercise and Sport Science, East Carolina University, Greenville, NC, USA; 5 Department of Radiology, Vanderbilt University Medical Center, Nashville, TN, USA; 6 Rheumatology Department, Kolling Institute, University of Sydney, Sydney, Australia; 7 Department of Physiotherapy, The University of Melbourne, Parkville, VIC, Australia; 8 Department of Radiology, Boston University School of Medicine, Boston, USA; 9 Section on Molecular Medicine, Wake Forest School of Medicine, Winston-Salem, NC, USA; 10 Department of Rheumatology and Immunology, Wake Forest School of Medicine, Winston-Salem, NC, USA

## Abstract

**Background:**

Muscle loss and fat gain contribute to the disability, pain, and morbidity associated with knee osteoarthritis (OA), and thigh muscle weakness is an independent and modifiable risk factor for it. However, while all published treatment guidelines recommend muscle strengthening exercise to combat loss of muscle mass and strength in knee OA patients, previous strength training studies either used intensities or loads *below* recommended levels for healthy adults or were generally *short*, lasting only 6 to 24 weeks. The efficacy of high-intensity strength training in improving OA symptoms, slowing progression, and affecting the underlying mechanisms has not been examined due to the unsubstantiated belief that it might exacerbate symptoms. We hypothesize that in addition to short-term clinical benefits, combining greater duration with high-intensity strength training will alter thigh composition sufficiently to attain long-term reductions in knee-joint forces, lower pain levels, decrease inflammatory cytokines, and slow OA progression.

**Methods/Design:**

This is an assessor-blind, randomized controlled trial. The study population consists of 372 older (age ≥ 55 yrs) ambulatory, community-dwelling persons with: (1) mild-to-moderate medial tibiofemoral OA (Kellgren-Lawrence (KL) = 2 or 3); (2) knee neutral or varus aligned knee ( -2° valgus ≤ angle ≤ 10° varus); (3) 20 kg^.^m^-2^ ≥ BMI ≤ 45 kg^.^m^-2^; and (3) no participation in a formal strength-training program for more than 30 minutes per week within the past 6 months. Participants are randomized to one of 3 groups: high-intensity strength training (75-90% 1Repetition Maximum (1RM)); low-intensity strength training (30-40%1RM); or healthy living education. The primary clinical aim is to compare the interventions’ effects on knee pain, and the primary mechanistic aim is to compare their effects on knee-joint compressive forces during walking, a mechanism that affects the OA disease pathway. Secondary aims will compare the interventions’ effects on additional clinical measures of disease severity (e.g., function, mobility); disease progression measured by x-ray; thigh muscle and fat volume, measured by computed tomography (CT); components of thigh muscle function, including hip abductor strength and quadriceps strength, and power; additional measures of knee-joint loading; inflammatory and OA biomarkers; and health-related quality of life.

**Discussion:**

Test-retest reliability for the thigh CT scan was: total thigh volume, intra-class correlation coefficients (ICC) = 0.99; total fat volume, ICC = 0.99, and total muscle volume, ICC = 0.99. ICC for both isokinetic concentric knee flexion and extension strength was 0.93, and for hip-abductor concentric strength was 0.99. The reliability of our 1RM testing was: leg press, ICC = 0.95; leg curl, ICC = 0.99; and leg extension, ICC = 0.98. Results of this trial will provide critically needed guidance for clinicians in a variety of health professions who prescribe and oversee treatment and prevention of OA-related complications. Given the prevalence and impact of OA and the widespread availability of this intervention, assessing the efficacy of optimal strength training has the potential for immediate and vital clinical impact.

**Trial registration:**

ClinicalTrials.gov, NCT01489462

## Background

By 2030, an estimated 67 million American adults will report physician-diagnosed arthritis—a 40% increase in 25 years [1]. Osteoarthritis (OA) is the most common form of arthritis and the leading cause of disability among adults; the prevalence of self-reported doctor diagnosed OA in the United States is estimated at greater than 27 million persons [2]. Knee OA accounts for a significant portion of this disability, and is largely due to factors that alter knee-joint loading. Results from this project will inform future management of patients suffering from knee OA and could have enormous public health implications.

Muscle loss and fat gain contribute to the disability, pain, and morbidity associated with knee OA [3], and thigh muscle weakness is an independent, modifiable risk factor [4,5]. While treatment guidelines recommend strengthening exercise to combat sarcopenia in knee OA patients [6,7], the appropriate intensities or loads (defined as percent of one repetition maximum, or %1RM) recommended are unclear. The intensities used in previous OA studies were *below* those recommended by the American College of Sports Medicine for healthy adults [8] (60-80% 1RM). Further, the programs were generally *short*, between 6 and 24 weeks [9-16]; effect sizes were low-to-modest, changes in progression could not be detected, and they provided little lasting clinical benefit. Indeed, short-term exercise benefits are gone 6 months post-exercise [17-19] but long-term supervised exercise results in sustained benefits 2 years after the treatment ends [20]. Few have studied the effectiveness of more intense strength training due to the unsubstantiated belief that it might exacerbate OA symptoms [21]. Preliminary studies indicate that high-intensity strength training is safe and well tolerated by healthy older adults [22,23] and knee OA patients [16,24].

Greater thigh fat is associated with obesity, a major risk factor for knee OA [25]. Obesity combined with sarcopenia, termed sarcopenic obesity, is also closely associated with the prevalence of knee OA with an Odds Ratio = 3.51 [26]. Intensive strength training can change thigh composition in older adults and has shown promise in treating the underlying biomechanical (knee-joint loading) and inflammatory disease pathways. Studies in healthy older adults associate intensive strength training with increased fat-free thigh mass and quadriceps cross-sectional area and decreased percent body fat and thigh subcutaneous fat with minimal alteration in total body weight [27-30]. Sipila and Suominen [27] and Ferri et al. [23] noted increased quadriceps cross-sectional area and lean cross-sectional area, and less intramuscular thigh fat after 16–18 weeks of intensive strength training. Similarly, Treuth et al. [31-33] found increased thigh muscle mass and decreased thigh fat mass after 16 weeks of high-intensity strength training in older men and women. High-intensity strength training also reduced interleukin (IL)-18, a pro-inflammatory cytokine, in HIV-infected patients [34] and IL-6 and C-reactive protein (CRP) levels in older adults with chronic kidney disease compared to controls [35]. We must now gather clinical and mechanistic evidence to determine if improved thigh muscle composition has *long-term protective effects* on joint mechanics, inflammation, and structural progression in knee OA.

**Figure 1:**
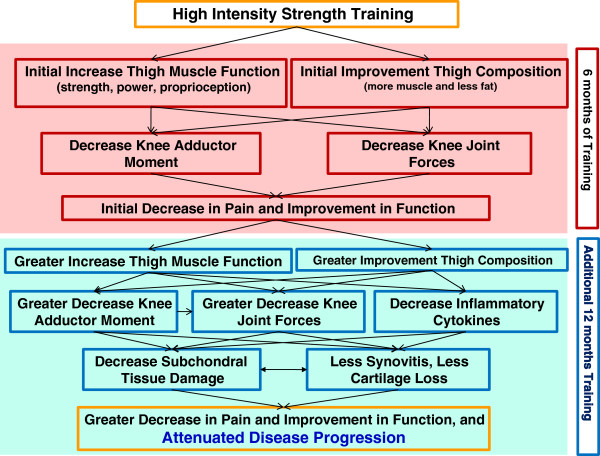
Hypothesized pathways mediating high-intensity strength-training outcomes at 6 and 18 mos.

This paper describes the design of the Strength Training for ARthritis Trial (START), the first long-term clinical trial comparing the efficacy of *high-* (75-90% 1RM) to *low-intensity* (30-40% 1RM) strength training and Healthy Living Education interventions in older adults with knee OA. This trial is designed to identify potential mechanisms, (i.e. knee joint loading and systemic inflammation) responsible for any changes in pain, function, and mobility consequent to intensive strength training. We expect initial improvements in thigh muscle function, pain, and knee-joint loading with high-intensity strength training after 6 months; 18 months will determine, *for the first time*, if further changes in thigh muscle function and composition significantly reduce knee-joint forces and inflammatory cytokines resulting in a greater decrease in pain and attenuated OA disease progression (Figure 1). Given the prevalence of OA, the detrimental effects of sarcopenia and obesity [26], and the safety and widespread availability of the intervention, this trial has immediate, potentially transformative clinical impact.

## Methods/Design

### Study design

START is an assessor-blinded, single-center, 18-month, parallel design randomized controlled trial. Participants are randomized into one of 3 groups: high-intensity strength training (H), low-intensity strength training (L), or healthy living education (C).

### Study sample

The study sample consists of 372 ambulatory, community-dwelling adults age ≥55 yrs with self-reported disability due to knee OA. Inclusion criteria include: (1) mild-to-moderate radiographic medial tibiofemoral OA (Kellgren-Lawrence (KL) = 2 or 3); (2) knee neutral or varus aligned knee ( -2° valgus ≤ angle ≤ 10° varus); (3) BMI ≥ 20 kg^.^m^-2^ and ≤ 45 kg^.^m^-2^; and (4) no participation in formal strength training for more than 30 min^.^wk^-1^ in the past 6 months. We exclude people with BMI >45 kg^.^m^-2^ because of difficulty in using CT equipment and lower adherence to exercise [36,37] and <20 kg^.^m^-2^ because of limited thigh fat. We include only people with neutral (−2° valgus to 2° varus) or moderate varus (≤ 10° varus) alignment and medial knee OA and *not* predominant lateral compartment or severe patellofemoral (PF) compartment disease because (1) the medial compartment is the most common disease site, and (2) medial progression is strongly associated with moderate varus alignment [38-40], independent of BMI [41]. People with extreme malalignment (>10 degrees varus) might experience greater progression in a strengthening program [21,42]. Medial bone-marrow lesions are seen mostly in patients with varus limbs, who are most likely to progress medially [43]. This approach will engage an enriched cohort of structural progressors to determine better our intervention’s ability to slow the disease [16]. All participants may maintain their medications, including NSAIDs. If pain decreases, they may reduce them with their physician’s consent. Medication use is recorded at baseline and 6-, 12-, and 18-month follow-up testing. Exclusion criteria are listed in Table 1. The study protocol was reviewed and approved by the Human Subjects Committee of Wake Forest University Health Sciences (Human Protocol: IRB00018176) and is in compliance with the terms and conditions set forth in the Helsinki Declaration (http://www.wma.net/en/30publications/10policies/b3/index.html). Informed consent will be obtained from all study participants.

**Table 1:** Exclusion criteria

**Criteria**	**Exclusion**	**Method**
Significant co-morbid disease that would threaten safety or impair ability to participate in interventions or testing, previous acute knee injury, bilateral severe tibiofemoral OA, severe patellofemoral OA (JSN = 3 using OARSI atlas), no definite medial tibiofemoral OA, severe obesity, low weight.	Symptomatic or severe coronary artery disease; severe HTN; active cancer other than skin cancer; anemia; dementia; liver disease; COPD; peripheral vascular disease; inability to walk without an assistive device; blindness; type 1 diabetes; type 2 diabetes on thiazolidinedione agents; bilateral severe medial tibiofemoral OA (KL = 4), no definite medial tibiofemoral OA (KL = 0, 1), BMI < 20 or > 45 kg^.^m^-2^	Medical history; physical exam; PA and skyline knee x-ray; height and weight.
OA disease location and alignment restrictions: predominant knee OA other than medial tibiofemoral OA; valgus, or extreme varus alignment.	Lateral tibiofemoral OA > medial tibiofemoral OA, severe patellofemoral OA; valgus knee alignment > 2°, or varus alignment > 10°	Knee PA and skyline view x-rays, lower extremity long x-ray.
Excess alcohol use	≥ 21 drinks per week	Questionnaire
Inability to finish 18-month study or unlikely to be compliant	Lives > 50 miles from site or planning to leave area ≥ 3 months during the next 18 months	Questionnaire, interview
Conditions that prohibit CT	BMI > 45 kg^.^m^-2^	height and weight
Significant cognitive impairment	diagnosis of dementia or a MoCA score <20	Medical history, MoCA
Low Pain	Pain ≤ 3 on a scale from 0-20	WOMAC

### Interventions

#### Strength training

Both strength training interventions consist of a 5-min warm-up, 40-min training, and 15-min cool-down. The 60-min sessions are conducted 3 times^.^wk^-1^ for 18 months at the Wake Forest Clinical Research Center. Each group session will include between 12–24 participants (1 to 2 waves) and will be supervised by two American College of Sports Medicine certified exercise interventionists and a number of undergraduate interns. The first two sessions introduce participants to proper techniques, and at the third, 1-repetition max (1RM) tests determine the starting resistance used for each exercise in the subsequent sessions until the next assessment (detailed below). Intensity (load) is defined as %1RM [8]. The reliability of our 1RM testing for 12 older adults with knee OA tested twice one week apart was: leg press, ICC = 0.95; leg curl, ICC = 0.99; and leg extension, ICC = 0.98. Each exercise is performed on a Nautilus resistance-training machine with 60–90 s of rest between sets; 1RM is defined as the maximum weight one can lift in a single repetition. Participants will keep a session log of each resistance exercise, its weight setting, and number of sets and repetitions achieved. Although our hypotheses focus on the lower extremity, experience indicates that participants want a well-rounded program. Thus, for both groups the program includes 6 lower body exercises with each leg exercised separately to prevent an unequal distribution of load between the least affected and most affected sides: hip abduction and adduction; leg curl, extension, and press; and seated calf; and 4 upper body and core exercises: compound row, vertical chest, lower back, and abdomen. We use Nautilus machines based on time, safety, and availability, but results will be generalizable to most strength training methods. Participants perform a 5 minute warm-up on either the walking track or a stationary bicycle and, at the completion of each strength training session, a 15 minute cool-down consisting of various upper and lower body stretching exercises.

Participants who plan absences of >2 sessions use Thera-Bands in a home-based program. Upon their return, interventionists determine the progression needed to reach prior intensity.

Previous strength training trials with older adults predict small fluctuations in body weight (< 1 kg) as muscle mass increases and fat mass decreases [44]. Interventionists are alert to any substantial change (≥ 2 kg) and, if necessary, the participant is referred to the medical director.

#### High-intensity intervention (H)

The H group performs 3 sets of each exercise at 75-90% of 1RM, within the intensity range necessary to maximize muscular hypertrophy [8]. Each block has the following structure and is repeated with training loads recalibrated to each new 1RM:

Weeks 1–2. 3 sets by 8 reps. Intensity: 75% of 1RM

Weeks 3–4. 3 sets by 8 reps. Intensity: 80% of 1RM

Weeks 5–6. 3 sets by 6 reps. Intensity: 85% of 1RM

Weeks 7–8. 3 sets by 4 reps. Intensity: 90% of 1RM

Week 9. Taper. Alternate exercises and 1RM testing

Most participants have no difficulty progressing at 2-wk intervals, but variation is inevitable. Participants will rate perceived exertion (RPE) at completion of each workout. On a 10-point Borg category ratio-RPE scale, the H group should be working between 5 (hard)-8 (very hard), and the L group between 2 (easy)-4 (somewhat hard) [45]. At the end of each block, we add taper periods—2 days (Monday, Friday) of alternate exercises using Thera-Bands, separated by a 1RM testing day (Wednesday)—because the use of Thera-Bands has been shown to increase performance in older women [46].

#### Low-intensity intervention (L)

The L group performs 3 sets of 15 repetitions at 30-40% of 1RM using the exercises described above. Each 8-week block has the following structure:

Weeks 1–2. 3 sets by 15 reps. Intensity: 30% of 1RM.

Weeks 3–4. 3 sets by 15 reps. Intensity: 35% of 1RM.

Weeks 4–6. 3 sets by 15 reps. Intensity: 40% of 1RM.

Weeks 7–8. 3 sets by 15 reps. Intensity: 35% of 1RM.

Week 9. Taper week. Alternate exercises and 1RM testing

Repeat weeks 1–8 with training loads recalibrated to each new 1RM.

The workloads for the H and L groups are equated (Table 2).

**Table 2:** Sample workloads and total volume for high- and low-intensity interventions, assuming 1RM = 100 lbs

**Intervention**	**Sets/Repetitions/Intensity**	**Volume**
**Low Intensity**		
Weeks 1-2	3 sets of 15 reps at 30% 1-RM	45 reps*30 lbs = 1350 lbs* 2 wks = 2700 lbs
Weeks 3-4	3 sets of 15 reps at 35% 1-RM	45 reps*35 lbs = 1575 lbs* 2 wks = 3150 lbs
Weeks 5-6	3 sets of 15 reps at 40% 1-RM	45 reps*40 lbs = 1800 lbs*2 wks = 3600 lbs
Weeks 7-8	3 sets of 15 reps at 35% 1-RM	45 reps*35 lbs = 1575 lbs* 2 wks = 3150 lbs
**Weeks 1 thru 8**		**Total volume = 12600 lbs**
**High Intensity**		
Weeks 1-2	3 sets of 8 reps at 75% 1-RM	24 reps*75 lbs = 1800 lbs*2wks = 3600 lbs
Weeks 3-4	3 sets of 8 reps at 80% 1-RM	24 reps*80 lbs = 1920 lbs*2wks = 3840 lbs
Weeks 5-6	3 sets of 6 reps at 85% 1-RM	18 reps*85 lbs = 1530 lbs*2wks = 3060 lbs
Weeks 7-8	3 sets of 4 reps at 90% 1-RM	12 reps*90 lbs = 1080 lbs*2 wks =2160 lbs
**Weeks 1 thru 8**		**Total volume = 12660 lbs**
		**Low/High Ratio = 1.0**

Total volume = total repetitions _*_ intensity _*_resistance (assume 100 lbs).

#### Healthy living education

The control group is modeled after the Arthritis Diet and Activity Promotion Trial’s (ADAPT) healthy lifestyle comparison group [47], providing attention, social interaction, and health education. Participants attend 60-min organized workshops bi-weekly for the first 6 months and monthly thereafter. This arm aims to control for attention from study staff and general levels of participant time; to encourage recruitment, adherence and benefit; and not to influence the primary outcomes directly: no evidence suggests that health education alone will affect pain or knee-joint loads during walking. The decision to use a tapered schedule is based on careful consideration of science, adherence, and expense. The control group is not matched hour-for-hour to the intervention groups but reflects the way community health-education programs are typically offered and the observation that older adults are less likely to attend more often.

Over the 18 months, interactive presentations cover such topics as hearing loss, nutrition, managing medication, and sleep practices, and experts will give wide-ranging lectures. An experiential component encourages participants to seek more information about their health and related practices. They are asked to complete homework, review topics, and engage in small group discussions to increase their involvement in this study arm. Each workshop ends with seated upper body stretching to enhance adherence and increase perceived benefit without directly affecting the knees or study outcomes. Prior studies suggest older adults are less likely to participate if they think any treatment group does not provide personal benefit.

#### Techniques to improve adherence and retention

Time-intensive behavioral studies require significant commitment [47-49]. START’s design evolved from social cognitive theory (SCT), group dynamics, and over 22 years’ experience in randomized controlled trials: our 18-month trials Fitness Arthritis and Seniors Trial (FAST), ADAPT, and Intensive Diet and Exercise for Arthritis (IDEA) had between 80%- 88% retention and 58-70% adherence. We estimate 80% retention and 65% adherence rates over the intervention; adherence is calculated by dividing the total number of sessions completed by the number scheduled.

START interventionists are trained by our health psychologist in standardized behavioral techniques developed in a SCT framework. They include frequent contact during the intervention; positive feedback; incentives to reach attendance and performance goals; establishing personal commitment to the project; and targeted mechanisms for behavioral adherence, including self-efficacy, outcome expectations, and self-regulatory skills. The importance of regular attendance is emphasized with study participants on an ongoing basis. Adherence data are reviewed regularly to identify any participants who need additional reminders and/or counseling. Our toolbox approach, guided by algorithms of common strategies and decision-making processes, tailors the intervention to each participant’s needs. For example, if a participant misses two consecutive sessions and has no contact with the interventionist, a phone session is scheduled. The interventionist assesses participant study goals and barriers to participation. Together, participant and interventionist develop a specific plan. Collectively, these strategies increase social cognitive mechanisms for regular participation and enhanced adherence in all groups.

### Trial conduct

#### Recruitment

The 30-month recruitment period is divided into 10 waves of approximately 37 participants each, entering the study at 3-month intervals. The predominant recruitment strategy is newspaper advertisements. Other recruitment efforts include mass mailings, presentations at local aging service networks, senior centers, churches, radio, and on-line advertising. Our Claude D. Pepper Older Americans Independence Center recruitment core also has access to a large database of older adults who have consented to be contacted about participation in clinical trials. Specific strategies aim to maximize the number of African Americans who qualify for, and are enrolled in, the study. At biweekly meetings, all recruitment activities and the number of participants randomized are reviewed.

### Measurements

#### Screening and follow-up visits

**Figure 2:**
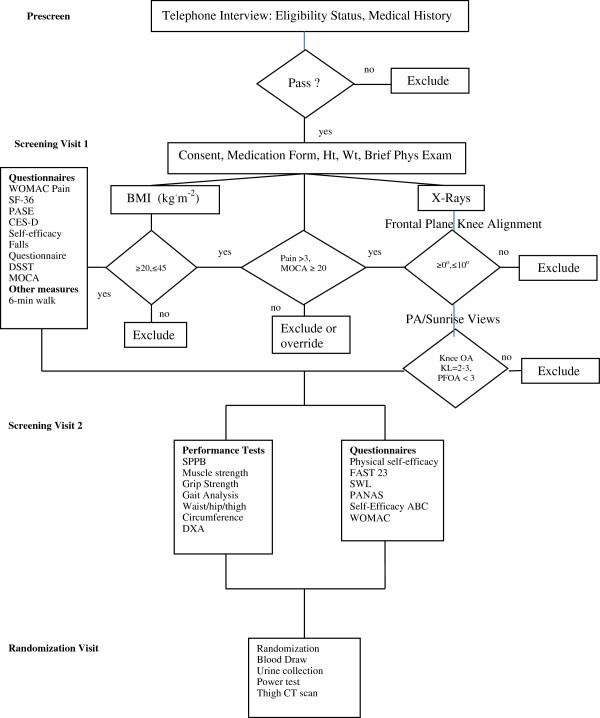
Participant eligibility and screening flow chart.

Those who are eligible after prescreening (PSV) sign an informed consent and attend 2 screenings (SV) and a randomization visit (RV). All 372 participants are measured at baseline, 6-, 12-, and 18-month follow-ups (FU) (Figure 2 and Table 3).

**Table 3:** Data-collection visits

**Measurements**	**PSV**	**SV1**	**SV2**	**RV**	**FU6**	**FU12**	**FU18**
randomization				x			
informed consent		x					
eligibility questionnaire	x						
medical history	xc	x			x	x	x
WOMAC		x	x		x	x	x
PASE scale		x			x	x	x
MoCA		x			x	x	x
CES-D		x			x	x	x
SF-36 (general health, quality life)		x			x	x	x
Self Efficacy		x			x	x	x
6-min walk		x			x	x	x
Demographics		x					
Brief physical exam		x					
Medication Form		x			x	x	x
Knee A-P x-ray		x					x
Knee x-ray skyline view		x					
Full length lower extremity x-ray		x					
height	xc	x					
weight	xc	x			x	x	x
Waist/Hip circumference			x		x	x	x
DXA			x				x
CT scans: thigh				x			x
Biomarkers: blood				x			x
Biomarkers: urine				x	x		x
Gait Analysis			x		x		x
Muscle function tests: Power, Strength			x		x	x	x

#### Screening

The Eligibility Questionnaire addresses joint pain, physical function, activity level, co-morbid diseases, willingness to participate for 18 months, height and weight (to determine BMI), caregiver status, status of significant others, and distance of home from the center (within 50-mile radius). A study physician and a physician assistant perform routine medical exams. A score of <20 on the Montreal Cognitive Assessment (MoCA) [50,51] will justify exclusion because cognitively impaired persons may not be able to adhere to the protocol; persons scoring >17 on the Center for Epidemiologic Studies Depression scale (CES-D) [52] are evaluated by the study physician, who determines eligibility.

#### Western Ontario McMasters Universities Osteoarthritis Index (WOMAC)

Self-reported pain (primary clinical outcome) and physical function are measured using the Likert version of WOMAC [53]. The pain index assesses participants’ pain on a scale, ranging from 0 (none) to 4 (extreme). The pain subscale consists of 5 items and total scores can range from 0–20, with higher scores indicating greater pain. This instrument is recommended by the Osteoarthritis Research Society International as the health status measure of choice for older adults with knee OA. It has been validated for use in orthopaedic and pharmacologic interventions [53,54]. The pain subscale will be used only as a screening tool during SV1 (pain must be > 3). It will also be administered to the eligible participants at SV2 and each scheduled follow-up visit.

For physical function, the Likert version asks participants to indicate on the same scale from 0 (none) to 4 (extreme) the degree of difficulty experienced performing activities of daily living in the last 48 hours due to knee OA. Individual scores for the 17 items are totaled to generate a summary score that can range from 0–68, with higher scores indicating poorer function.

#### Gait

The primary mechanistic outcome is maximal knee compressive force; secondary outcomes include internal knee abduction moment and AP shear force [55-59]. A 25-reflective marker set, 6-camera Motion Analysis System (100 Hz), and 2, 6-channel force plates (AMTI, Newton, MA. 1000 Hz) obtain 3D kinematic and kinetic gait data. The former will be acquired using Cortex 3.0 software (Motion Analysis Corporation, Santa Rosa, CA) and a Butterworth low-pass filter (6 Hz cutoff). For each participant, 3 successful trials are analyzed; i.e., within ±3.5% of the participant’s freely chosen speed, and the entire foot must contact the force plate in a visually normal stride. Smoothed coordinate data, ground reaction, and gravitational and inertial forces will inform an inverse dynamics model to calculate 3D moments and forces at the hip, knee, and ankle joints using Visual 3D Standard 4.0 clinical gait analysis software (C-Motion, Germantown, MD). These moments and forces will be used in the knee model developed by DeVita et al. [60] for use in knee OA subjects [58,59]. Model-predicted knee-compression force was also a primary outcome in IDEA. Our test-retest reliability intraclass correlations (ICC) for 21 knee OA patients with mean age 65.7 yrs (SD = 5.8) were r = 0.86 for internal peak knee extensor moment, r = 0.94 for internal peak abductor moment, and r = 0.95 for peak knee compressive force [61]. A detailed explanation of our model can be found elsewhere [62].

**Figure 3:**
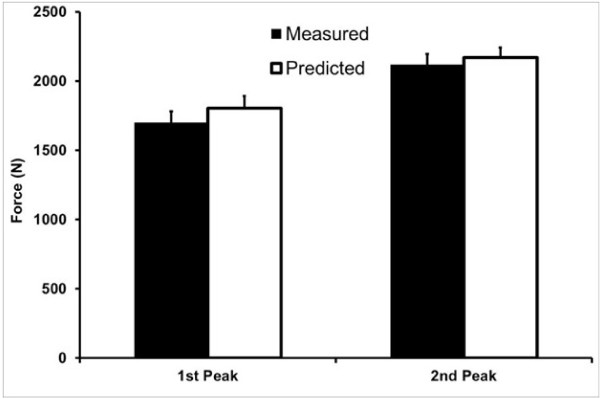
Comparison of peak knee compressive forces derived from our musculoskeletal model (predicted) using Fregly et al. data as input, and the measured value from an instrumented prostheses (measured) of the same subjects.

Numerous biomechanical-neuromuscular models exist that predict knee joint forces during locomotion. Many of these models predict highly similar results, strengthening the confidence researchers have in these models [63-65]. True biomechanical model validation is difficult and most predicted results have not been compared to measured knee joint forces, a gold standard for model validation. Recently, Fregly et al. [66] have made available measured *in vivo* knee joint force data during walking along with all pertinent biomechanical data for the purpose of validating biomechanical models predicting knee joint loads. These data come from individuals with instrumented knee joint prostheses and are available through the website, https://simtk.org/home/kneeloads. We used their biomechanical gait data as input for our model to predict knee joint forces from five walking trials and then compared our results to Fregly’s actual measured values from the same subjects. Our predicted values were highly similar to the measured values with the predicted first and second maximum compressive forces within 7% and 3% of the observed values, respectively (Figure 3).

#### Mobility

Our measure of mobility is 6-min walk distance, with a 3-month test-retest reliability of 0.86 [67].

#### Health-Related Quality of Life (HRQL)

The SF-36 [68] is the most widely used and carefully validated measure of HRQL. It yields two broad summary scores: physical health and mental health.

#### Body composition

Dual-energy x-ray absorptiometry (DXA) (GE Healthcare iDXA Digital Densitometry) is used for measurements of bone mineral density (BMD) and body tissue composition including whole body changes in total fat (FM) and lean (LM) mass [69-71]. Percent coefficients of variation (%CV) are 1.2% for whole body FM; 0.5% for whole body LM; 0.9% for whole body BMD; 1.2% for posterioranterior (PA) spine BMD; and 0.9% for total hip BMD.

#### Thigh composition

A secondary outcome is the measurement of thigh skeletal muscle and adipose tissue using volumetric non-contrast enhanced computer tomography (CT) (VCT 64, GE Healthcare Waukesha, WI). Participants will be placed supine on the CT couch with their legs held in a neutral position by dedicated Velcro straps. A calibration phantom (Image Analysis, Columbia, KY) with known CT densities for fat, water, and calcium is placed in the scan fields of view (FOV) posterior to the legs. A 2-dimensional topogram is obtained covering from the pelvis to the knees. A single helical scan series is performed starting at the femoral head and ending below the knee joint in the tibial plateau. Technique for the scan includes: helical mode, 120 KV, 150 mAs, with reconstruction of both legs at 5 mm slice thickness and 50 cm display field of view (DFOV). Additional reconstructions are performed using the same exposure to the participant to obtain sets of images using thinner slices (1.25 mm and 0.625 mm) using both standard and bone kernels to optimize image quality. Each femur has a set of high-resolution 30-cm dfov targets for potential future analysis of cortical bone structure.

#### CT image analysis software and protocol

The topogram of the femur is measured from the greater tuberosity of the femur to the inferior aspect of the medial femoral condyle. This length is trisected, and the junction between the proximal and mid-third is the landmark for measurement. Measurements of right and left thigh composition are performed using the slices that start at the location 25 mm above and the end 25 mm below this point, providing a sampling length of 50 mm along the long axis of the femur (e.g. head to foot). CT images are analyzed (reader masked to treatment group) on a medical imaging workstation with custom plugins to measure the volume of the entire thigh, thigh musculature, and intermuscular fat. Test-retest reliability on the thigh CT scans of a quality control sample of START participants (n = 10) re-analyzed an average of 3.7 months apart was: total thigh volume, ICC = 0.99; total fat volume, ICC = 0.99, and total muscle volume, ICC = 0.99.

#### Blood and urine sample collection

Blood samples (50 ml per visit) for assessing biomarkers are collected via venipuncture at a specific time in the morning at least 2 hours after rising and after a *10-hour overnight fast* at baseline and at 6- and 18-month assessment visits. Urine samples (second am void, 20 ml per visit) are collected in 250 ml specimen cups by each participant for analysis of new and emerging OA biomarkers. Serum, plasma, and urine are aliquoted and stored at -80°C until analysis of our pre-chosen biomarkers (see below) at the end of the study. Aliquots are also stored long-term to test for promising new inflammatory and OA biomarkers that may become available after the study.

#### Inflammatory markers

IL-6, tumor necrosis factor-alpha (TNFα), and soluble TNF receptor 1 (sTNFR1) were chosen for their known implication in OA [72-77]. They have been shown to change with 1 year of moderate strength training [78]. *IL-6 is our primary inflammation measure.* All inflammatory marker assays are performed in the WFSM ELISA Core Laboratory. All samples are measured in duplicate, using the average for analyses. Commercially available (R&D Systems, Minneapolis, MN) enzyme-linked immunosorbent assay (ELISA) kits are used: high-sensitivity Quantikine® for IL-6. In our laboratory, inter- and intra-assay coefficients of variation (CV) for IL-6 are 5.4% and 3.5%, respectively; for TNFα, 11.8% and 6.2%, respectively; and under 5% for the soluble receptor assays

#### OA Biomarkers

There is a lack of gold standard biomarkers for OA and so the choice of biomarkers that will be measured will be based on the most recent findings available at the completion of the trial. Possible markers could include serum COMP (AnaMar Medical, Uppsala, SW) and urine markers of collagen degradation such as C2C-HUSA (Ibex Technologies, Montreal, CN).

#### Muscle function tests

##### Strength

Knee flexion/extension concentric strength is assessed at baseline, 6, 12, and 18 months using a Humac NORM isokinetic dynamometer (Computer Sports Medicine Inc, Stoughton, MA) set to 30 deg^.^s^-1^. Strength test-retest reliability for 10 participants tested twice in our lab, 7–10 days apart, had an ICC of 0.93 for both concentric knee flexion and extension strength. Since we suggest that intensive strength training can reduce knee-joint loads by counterbalancing the external knee-adductor moment with strong hip abductors, we also measure hip-abductor concentric strength at 30 deg^.^s^-1^ (ICC = 0.99). Knee strength is measured with the participant in a seated position; hip abductor strength is measured in a side lying position.

##### Power

The Nottingham power rig is used to measure bilateral leg extensor power because it correlates well with such functional measures as chair-rise, stair-climbing, and walking speed in elderly subjects [79]. This measurement is safe and acceptable for all age groups [79,80].

#### Medications

A questionnaire adapted from the Atherosclerosis Risk In Communities (ARIC) [81] study and widely used in field research and our studies is designed to obtain information (during SV1 and FU visits) about all prescription and over-the-counter medicines and supplements used during the 2 weeks prior to interview.

#### X-ray

Bilateral PA weight-bearing knee x-rays using a positioning device (SynaFlexer™, Synarc Inc., San Francisco, CA) and the modified Lyon-Schuss technique [82] are used to identify tibiofemoral (TF) OA and skyline views to identify PF OA. The former is repeated at 18 months to assess changes in joint-space width (JSW). Radiographs are evaluated using the K-L score of 0–4 to assess disease severity as we have described and we include only participants with a score of 2 or 3 in at least one knee (KL score = 4 is acceptable in the contralateral knee). We exclude people with severe PF OA (JSN = 3 on a 0–3 scale using the OARSI atlas) and control for severity (none-to-moderate) in statistical analyses. We define medial TF disease based on our previous definitions [83], and participants with lateral > medial joint space narrowing are excluded.

The knee PA protocol includes positioning the participant standing and facing the Bucky or cassette holder with the x-ray beam centered on the joint line angulated caudally 10 degrees. The x-ray is repeated at 5 and 15 degrees and the most acceptable image (based upon alignment of the medial tibial plateau) is used for analysis. Criteria include good contrast/exposure, optimal visualization of the articular surfaces with the floor of the medial tibial plateau clearly delineated, and both knee joints included and centered on the film.

To assess alignment, a full-length AP radiograph of each lower extremity is obtained at baseline with participants positioned following Sharma et al. [84]. Mechanical alignment is the measure of the angle formed by the intersection of the lines connecting the centers of the femoral head and intercondylar notch and the centers of the ankle talus and tibial spines, with neutral angles between 0-2° varus, varus knee angles >2° inward, and valgus angles >0° outward*. The range of eligible knee angles is -2°valgus to 10*° *varus.*

Disease progression is defined as change in x-ray medial TF JSW. A physician, masked to treatment group, measures JSW using automated computerized software. The software automatically delineates the joint space contour with the help of an edge-based algorithm. The area of measurement of minimum JSW is defined by 2 vertical lines and 2 horizontal lines obtained by a single click on the nonosteophytic outer edge of the medial femoral condyle and a single click on the inner edge of the medial tibial plateau close to the articular surface. Within these landmarks, the delineation of the bone edges of the medial femoral condyle and medial tibial plateau floor, in addition to the minimum JSW, are automatically obtained [85].

#### Additional questionnaires

Physical Activity Scale for the Elderly (PASE) [86], Center for Epidemiologic Studies Depression Scale (CES-D) (92), and a self-efficacy for adherence measure [87] are used to assess physical activity, depression, and self-efficacy at baseline and follow-up. The Montreal Cognitive Assessment (MoCA) is used to measure cognitive functioning at baseline; a score ≥ 20 is required for study inclusion. The Digital Symbol Substitution Test (DSST) will be used to measure cognitive functioning.

#### Adverse event collection and reporting

An adverse event (AE) is defined as any unfavorable and unintended diagnosis, sign, symptom, or disease temporarily associated with the study intervention, which may or may not be related to the intervention. Non-serious AEs will be reported by the study staff to the project manager and principal investigator within 7 days of notification of the event. The study physician will review each non serious AE on a weekly basis that will be included in the NIAMS safety officer report that is submitted semi-annually. A serious AE (SAE) is any untoward medical occurrence that results in death, is life-threatening, requires or prolongs hospitalization, causes persistent or significant disability/incapacity, results in congenital abnormalities, or represents other significant hazards or potentially serious harm to research participants or others. Study staff will report SAEs to the project manager, study physician, and the principal investigator within 24 hours of notification. NIAMS will be notified within 24 hours subsequent to notification of the principal investigator.

### Timeline

Recruitment for each of 10 waves takes 3 months with an average of 37.2 participants/wave; an average of 12.4 participants are randomized/month for 30 months.

### Randomization

A stratified block randomization with block size unknown to investigators and staff ensures equal accrual to each study arm. Prestratification balances pretrial BMI values (20.0-24.9, 25.0-29.9, 30.0-34.9, 35.0-39.9, 40.0-45.0 kg^.^m^-2^) and gender, which could predict intervention effect and associations between secondary outcome variables.

### Data management

Data are collected on hard copy forms and transformed to an electronic database. We use a web-based management system to assure integrity and validity. Dynamic reports and periodic statistical analyses monitor quality. A participant-based inventory system tracks recruitment, retention, adherence, and missing data from entry through exit, close-out, and lock-down of final datasets. Our team developed a similar database for the IDEA study.

### Statistical considerations

#### Statistical analyses

All primary analyses are based on the intention-to-treat method in which each participant is included in the initial randomization group regardless of adherence. START stratification factors, baseline BMI, and gender are included in all statistical models, so the analysis matches the design, and the estimated variance is not biased. Assumptions are verified for all models, and appropriate transformations used when necessary.

##### Primary aim

The primary aim is the treatment effect on knee pain and maximal compressive force at 18 months. These and all other repeated measures continuous outcomes will be assessed using a mixed effects model including time (6 and 18 months), treatment group, the time × treatment interaction, and further adjusted for gender, baseline BMI, and baseline values of the outcome (i.e., baseline knee pain for knee pain model, baseline compressive force for compressive force model). The treatment effects at 18 months are tested by applying contrast statements to the mixed model, and maximum-likelihood techniques will be used to estimate parameters under the assumption of an AR(1) covariance structure. Each primary outcome will be analyzed at the Bonferroni-adjusted 0.025 level of significance, and pairwise comparisons between intervention groups at 18 months will use a 0.0083 two-sided level of significance (2 outcomes, 3 interventions) [88]. Preliminary analyses are conducted to check the shape of the distributions and variances between groups and as a function of the covariates to ensure residuals are approximately normally distributed. Regression diagnostics and residual plots help find appropriate transformations if necessary. In subsequent models, we will control for possible confounders, including PF OA severity (none to moderate) and use of medications, such as analgesics, NSAIDs, bisphosphonates, and glucosamine/chondroitin sulfate. Because we exclude subjects with severe PF OA and medication has only modest efficacy in OA, we do not expect significant confounding by these variables.

##### Secondary aims

Standard repeated measures mixed models (including adjustment variables as noted above) are used for secondary aims at the 0.05 significance level, with pairwise treatment group comparisons performed at the 0.0167 significance level for testing 18 month effects. Short-term effects are determined by using contrast statements comparing 6-month treatment effect means from primary and secondary outcome models. Outcomes assessed at baseline and 18 months only (MoCa, DXA measures, and Knee PA x-ray) will be compared using an ANCOVA model for treatment effects adjusting for baseline BMI, gender, and baseline values of the outcome.

As inflammatory marker distributions are often skewed, data are log-transformed before analysis. The effect of the interventions at 18 months is determined with repeated measures mixed models and estimates obtained at each visit. For ease of interpretation, transformed log means and standard errors back to their original units are used.

##### Missing data

If missing data are related to outcomes, our results will be slightly biased. Our models include variables from previous visits determined to predict loss to satisfy Little and Rubin’s [89] conditions for data considered Missing at Random (MAR). If “informative censoring” occurs, we will compare analyses using participants with complete data, multiple imputations, or explicit modeling of the censoring mechanism [90,91].

#### Sample-size calculations

##### Primary outcomes

A total sample of 372 (124/group) provide 80% statistical power to detect differences ≥17.6% in pain and ≥9.6% in maximal compressive force at the 2-sided 0.0083 significance level with 80% retention (2-sample *t*-test, Nquery Advisor). Standard deviations for pain and maximal compressive force were obtained from the START pilot (unpublished) and ADAPT [58,59] which measured the same outcomes and used similar patient populations; with mean differences of 1.12 (18%) and 657 N (20%), respectively.

##### Secondary outcomes

Overall, our sample size provides a moderate effect size of 0.46 with relevant detectable differences. ADAPT’s largest effect on WOMAC function was a 17% relative decrease. We are able to detect differences in thigh-muscle volume smaller than 10%. Goopaster [92] showed that after 12 months mean thigh-muscle attenuation significantly decreased by 1.4 HU in a control group but not in a physical activity group. With 6 more months of physical activity, we expect to detect group differences as small as 2.3 HU. A magnitude of 22% reduction in IL-6 is similar to reductions seen in clinical trials of statins and other anti-inflammatory medications [93-98].

## Discussion

Despite strong evidence for the potent effect of mechanics on disease progression and symptoms [55,99], there are few interventions that target mechanical load. One such intervention is strength training. However, many still believe that strength training for knee OA in general, and high-intensity strength training specifically, may exacerbate knee pain and be deleterious to joint structure. It is critical to evaluate the potential benefit (or harm) of commonly used therapeutic interventions such as strength training. Due to conflicting data it is unclear if an increase in the total joint reaction force occurring with muscle strengthening contraction may actually *accelerate* the progression of structural changes within the joint, rather than prevent it [100]. A longitudinal study of 79 women with radiographic knee OA found that the mean absolute quadriceps strength of women with progressive OA (defined as worsening of the Kellgren and Lawrence grade over 2.5 years) was about 9% lower than those with radiographically stable OA [101]. In another observational study of 171 knee OA participants over 18 months, Sharma and colleagues [21] found that *greater* absolute quadriceps strength at baseline increased the risk of disease progression (defined as an increase in the grade of joint space narrowing in the medial or lateral compartment) in people with malaligned and lax knees (defined as >5° deviation from the mechanical axis) but not in those with neutral alignment. Since strength was not normalized for body mass, it is conceivable that the stronger participants were heavier, since absolute strength generally increases with body mass. More recently, Amin et al. [102] found no relationship between quadriceps strength and cartilage loss on MRI over 15 and 30 months anywhere except the lateral compartment of the patellofemoral joint, where increased quadriceps strength was protective against cartilage degeneration. Mikesky et al. [13] demonstrated a trend (p = 0.09) that strength training slowed joint space narrowing as measured on x-ray over 30 months in people with knee OA. It is important to recognize that they did not do intense training and did not elicit strength gains, just less strength loss, so different effects might be found in our study. Given the conflicting nature of the published literature and the public health impact of both a negative and a positive finding, the effects of strength training on pain, joint loading, and structure are critical to delineate.

There are several limitations and risks to our study. Our musculoskeletal model will estimate the knee joint compressive and shear forces. The principal limitations of most models are many simplifying assumptions about joint properties and structures that do not account for in-vivo symptoms and processes [39]. We have used our model extensively [58-60,103], and while it only estimates knee-joint biomechanics, the predictions for knee muscle and joint forces compare favorably with those of other predictive models, and are highly similar to measured forces from instrumented knee joint prostheses [63,66,104,105]. PF OA may also confound results. The symptoms in knee OA are frequently related to structural alterations in the PF joint, but despite great focus on the PF joint this relationship is relatively weak [106-108]. We will exclude people with severe PF OA (JSN = 3, OARSI scale) and control for severity (none to moderate) in statistical analyses. Also measures of JSN by plain films may not be sensitive enough to detect differences in radiographic progression over 18 months unless the differences are large [109].

Risks to participants are small. Musculoskeletal injury may occur as the result of the exercise intervention, but during the strength-training portion of the recent IDEA trial of 454 overweight or obese subjects with knee OA, we had no serious injuries. We will include a blood pressure safety alert trigger for this study. The absolute contraindication to resistance training is set at greater than 180/110 mmHg, and the relative contraindication at above 160/100 mmHg. Bilateral volumetric measures of thigh adipose tissue and skeletal muscle will use a standard CT protocol. The thigh sequence is centered on the mid-thigh and is about 33% of the expected exposure of a clinical scan of this region. The average amount of radiation a person will receive is low, 3 mSv (range 1.5-6 mSv). This value can be comparable to the U.S. average annual exposure from natural sources of 3 mSv and lower than the 7 mSv exposure of residents of Denver. The risk is comparable to, or less than, other risks encountered in daily life, such as driving or riding in a motor vehicle [110].

Given the prevalence and impact of OA and the widespread availability of strength training, assessing its efficacy has immediate and vital clinical impact. Results of this study will document accurately the effects of both high-and low-intensity strength training on knee joint pain, joint loads, inflammation, thigh composition, and disease progression, and provide critically needed guidance to clinicians who prescribe and oversee treatment and prevention of OA-related complications.

## Competing interests

The authors declare that they have no competing interests.

## Authors’ contributions

SPM conceived the study, participated in its design and coordination, carries out the biomechanical gait and strength analysis, and drafted the manuscript. SLM participated in its design, and coordinates patient compliance and adherence protocols. DPB participated in its design, coordinates statistical analyses and data management. BJN participated in its design and coordination, and carries out the biomarker analyses. PD participated in its design, helps coordinate the biomechanical gait analysis, and musculoskeletal modeling. JJC participated in its design, coordinates the CT scans and analysis, and helps coordinate the x-ray exams. DJH participated in its design and helps to coordinate x-ray and CT scan analyses. JDW participated in its design and is the medical director of the trial. KLB participated in its design and helps coordinate the strength interventions and testing. AG designed the x-ray protocol and analysis of joint space width. ML carries out patient evaluations. RFL participated in its design, and coordinates x-ray reading, and carries out osteoarthritis biomarker analysis. All authors read and approved the final manuscript.

## Pre-publication history

The pre-publication history for this paper can be accessed here:

http://www.biomedcentral.com/1471-2474/14/208/prepub
